# Microbial Community-Level Physiological Profiles and Genetic Prokaryotic Structure of Burned Soils Under Mediterranean Sclerophyll Forests in Central Chile

**DOI:** 10.3389/fmicb.2022.824813

**Published:** 2022-04-28

**Authors:** Humberto Aponte, Tania Galindo-Castañeda, Carolina Yáñez, Martin Hartmann, Claudia Rojas

**Affiliations:** ^1^Laboratory of Soil Microbial Ecology and Biogeochemistry (LEMiBiS), Institute of Agri-Food, Animal and Environmental Sciences (ICA3), Universidad de O’Higgins, San Fernando, Chile; ^2^Center of Applied Ecology and Sustainability (CAPES), Santiago, Chile; ^3^Sustainable Agroecosystems, Department of Environmental Systems Science, ETH Zurich, Zurich, Switzerland; ^4^Institute of Biology, Pontificia Universidad Católica de Valparaíso, Valparaíso, Chile

**Keywords:** rhizosphere, bacteria, Biolog EcoPlates, ecosystem recovery, wildfires

## Abstract

Forest fires alter soil microbial communities that are essential to support ecosystem recovery following land burning. These alterations have different responses according to soil abiotic pre- and post-fire conditions and fire severity, among others, and tend to decrease along vegetation recovery over time. Thus, understanding the effects of fires on microbial soil communities is critical to evaluate ecosystem resilience and restoration strategies in fire-prone ecosystems. We studied the state of community-level physiological profiles (CLPPs) and the prokaryotic community structure of rhizosphere and bulk soils from two fire-affected sclerophyll forests (one surveyed 17 months and the other 33 months after fire occurrence) in the Mediterranean climate zone of central Chile. Increases in catabolic activity (by average well color development of CLPPs), especially in the rhizosphere as compared with the bulk soil, were observed in the most recently affected site only. Legacy of land burning was still clearly shaping soil prokaryote community structure, as shown by quantitative PCR (qPCR) and Illumina MiSeq sequencing of the V4 region of the 16S rRNA gene, particularly in the most recent fire-affected site. The qPCR copy numbers and alpha diversity indexes (Shannon and Pielou’s evenness) of sequencing data decreased in burned soils at both locations. Beta diversity analyses showed dissimilarity of prokaryote communities at both study sites according to fire occurrence, and NO_3_^–^ was the common variable explaining community changes for both of them. Acidobacteria and Rokubacteria phyla significantly decreased in burned soils at both locations, while Firmicutes and Actinobacteria increased. These findings provide a better understanding of the resilience of soil prokaryote communities and their physiological conditions in Mediterranean forests of central Chile following different time periods after fire, conditions that likely influence the ecological processes taking place during recovery of fire-affected ecosystems.

## Introduction

Fires are key events regulating the structure and function of ecosystems (Yang et al., 2020); they have been important drivers of vegetation evolution and adaptation in most of the Mediterranean biomes across the globe. However, in the case of the Mediterranean ecosystem of central Chile, as opposed to the other four biomes of this type across the globe, land burning has not been considered an evolutionary force to select for fire-prone vegetation (Rundel et al., 2016, 2018). Fires have also represented an important pressure inducing land degradation worldwide, especially in ecosystems of warm and dry summers (Esteves et al., 2012), which are highly susceptible to the effects of climate change (Fletcher and Zielhofer, 2013). This is the case of Mediterranean ecosystems, which, despite occupying only about 2.2% of the land surface worldwide (Rundel et al., 2018), are considered biodiversity hotspots constantly threatened by anthropogenic disturbances (García-Vega and Newbold, 2020). This is of particular concern in Chile, where fire events are expected to increase due to extended megadroughts synchronized with human interventions (Fuentes-Castillo et al., 2019).

In forests, land burning not only exert an individual impact on each of their biotic and abiotic components but also on the relationship among these constituents, compromising the functionality of the whole ecosystem (Brown and Smith, 2000). Land burning can alter forest belowground conditions that are essential to support aboveground life (Certini, 2005). Several studies have reported a greater sensitivity of soil biological properties as compared with physicochemical properties to fire occurrence (Hart et al., 2005; Mataix-Solera et al., 2009). For microbial indicators, phylogenetic community structure has been shown to evidence greater sensitivity to land burning than those targeting soil ecosystem functions such as microbial biomass, respiration, and specific enzyme activities related to C, N, and P cycles (Pérez-Valera et al., 2019). The impact on soil microbial status is of particular importance since microbe-mediated soil processes are one of the most important drivers of ecosystem recovery in fire-affected lands (Neary et al., 1999). Forest fires can threaten soil organisms due to their different sensitivity to soil heating (Hart et al., 2005; Mataix-Solera et al., 2009; Rodríguez et al., 2018). In addition, indirect effects of fires occur by altering abiotic factors known to shape microbial community structure, such as soil pH (Lauber et al., 2009; Rousk et al., 2010), soil organic matter (SOM) and soil organic carbon (SOC) (Eilers et al., 2010), soil oxygen (e.g., aeration) (Fierer, 2017), and nutritional conditions (Lauber et al., 2008), all of which are affected by fires (Hart et al., 2005). Direct and indirect forest fire effects on soil microbiota can reduce the total number of microbial species (Certini, 2005), increase the proportion of bacteria over fungi (Fultz et al., 2016), and change the abundance of particular taxonomic groups (Pressler et al., 2019). Moreover, several studies have shown negative effects of land burning on soil microbial activity and functional diversity (Mataix-Solera et al., 2009; Wang et al., 2016; Alcañiz et al., 2018; Sadeghifar et al., 2020).

Shifts in prokaryote community composition following fires have been reported in Mediterranean ecosystems, which have been related to local environmental conditions prior to land burning (Goberna et al., 2012). Soil prokaryote communities play key soil functions, including soil weathering, primary production, and organic matter decomposition, all of which are essential in the ecological functioning of soils and susceptible to fires (Pérez-Valera et al., 2019). For example, increases in Proteobacteria and Firmicutes, and a decrease in Acidobacteria in Mediterranean burned soils after 2 and 3 years of fire have been previously reported (Rodríguez et al., 2018). Proteobacteria are recognized by their ability to respond to labile C sources particularly in the rhizosphere, while Acidobacteria have the ability to degrade cellulose and lignin (Lagos et al., 2015). Therefore, changes in soil bacteria composition induced by fires and alterations of soil abiotic properties will have a direct effect of soil ecosystem functions (Pérez-Valera et al., 2019). Although several studies have reported effects of forest fires on microbial composition at phylum level, the evaluation of such effects on Mediterranean ecosystems of central Chile is rare, especially when considering potential relationships with soil functionality and physicochemical properties after fire.

The degree of alterations in soil microbial community structure and functional diversity after fires greatly depends on soil physicochemical characteristics prior and after land burning, fire severity, vegetation type, and other site-specific conditions (Certini, 2005; Hart et al., 2005; Mataix-Solera et al., 2009). The effect of forest fires on soil microbial conditions also depends on time after fires, as immediate to long-lasting effects have been reported (Mataix-Solera et al., 2009; Singh et al., 2017). Fire perturbance to belowground conditions is usually expected to last until vegetation recovers over ecological successions (Hart et al., 2005). Soil microbial activity following fires in Mediterranean forests have been shown to increase within the first year of fire occurrence and then reach values comparable with unburned conditions after 32 months of land burning (Bárcenas-Moreno et al., 2011). Other studies have evidenced different responses of microbial activity indicators following fires, which mainly remain altered within 1 year after fires to later resemble unburned conditions over a 3-year time frame (Sadeghifar et al., 2020). In boreal forest ecosystems, bacterial community composition and diversity have also been shown to be affected following 1 year of land burning and then recovered after 11 years (Xiang et al., 2014). As the literature suggests, ecosystems affected by fires take varying time spans to recover after land burning. Nonetheless, it is known that changes in soil microbial conditions and ecosystem functioning rapidly occur within the earliest stages following fires; thus, studies focusing on post-fire dynamics in the scale of months to years are of particular interest to understand recovery in relation to plant succession and changes in soil properties (Knelman et al., 2017; Pérez-Valera et al., 2019). This is particularly true for understudied environments that are less represented in the literature where local evidence is required, as it is the case for fire-affected soils of the Mediterranean ecosystem of central Chile.

The present work aimed to evaluate the state of community-level physiological profile and prokaryotic community structure of soil compartments (bulk and rhizosphere soils) at two fire-affected sclerophyll forests in the Mediterranean zone of central Chile. These sites had similar arboreous plant composition but contrasting land location and time elapsed after fire (17 months and the other 33 months after fire occurrence). We hypothesized that fire occurrence affects community-level physiological profiles and prokaryote community structure to a different extent depending on the time elapsed after fires, with the least recent fire affected soils closely resembling unburned conditions. To accomplish this, we used microbial community-level physiological profiles to evaluate carbon source utilization patterns, molecular approaches such as qPCR, and high-throughput sequencing of 16S rRNA genes to assess abundance and structure of the prokaryotic community, and classical soil physicochemical analyses to describe microbial habitats.

## Materials and Methods

### Research Sites

**FIGURE 1:**
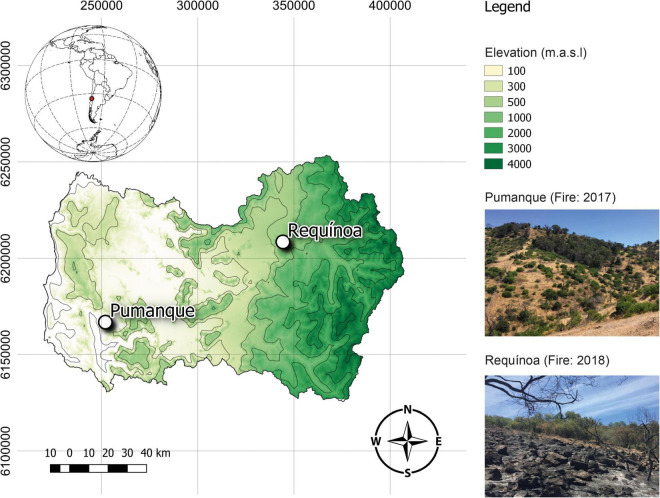
Sampling sites consisting of sclerophyll forests located within the Mediterranean climate zone of Central Chile, in the O’Higgins administrative region. White dots represent the study locations, which were affected by forest fires in January of 2017 (Pumanque) and May of 2018 (Requínoa). Image created using QGIS 3.12.3.

This study was conducted within the Mediterranean climate zone of Central Chile, specifically in the O’Higgins administrative region, at two locations affected by one forest fire event, either in January of 2017 or May of 2018 (Figure 1). The first research site (affected by a low-severity fire in January of 2017; García-Carmona et al., 2021) is in the Pumanque commune (34°35′44.99″ S; 71°42′17.349″ W) toward the east front of the coastal mountain range at an elevation of 100 m. At this place, a mean monthly precipitation of 36.7 mm is registered for the period 2000–2017 (Center of Climate and Resilience Research, n.d.). Soils are classified as Aquic Dystric Xerochrepts (Inceptisol), which originated from alluvium-colluvial parent materials, exhibited mainly loam to loamy sand textures, are stratified with 50–120 cm of depth, and have a mean slope of 15% (CIREN, 1996). The second site [affected by a low-severity fire in January of 2018, according to visual inspections recommended by Keeley (2009)] is in the Requínoa commune (34°14′19.314″ W, 70°41′26.52″ S) on the Piedmont of the west side of the Andes Mountain ridges at an elevation of 1,000 m. Here, a mean monthly precipitation of 25.0 mm was registered for the period 2000–2017 (Center of Climate and Resilience Research, n.d.). Soils at this place are classified as Mollic Haploxeralfs (Alfisol); they were developed on volcanic material, and exhibit loamy sand textures and shallow depths (20–80 cm depth), with a mean slope of 17% (CIREN, 1996). Vegetation at both sites is dominated by typical woody native species of the sclerophyll forest of Central Chile, including the tree species *Peumus boldus*, *Lithraea caustica*, and *Quillaja saponaria* and shrubs such as *Trevoa trinervis* (Supplementary Figure 1A). In addition, the herbaceous vegetation is composed by approximately 18 species in Requínoa and 30 species in Pumanque, of which six are common for both sites [*Erodium* sp., *Loasa triloba*, *Bromus hordeaceus*, *Hypochaeris radicata*, *Anthriscus caucalis*, *Oxalis micrantha* (Supplementary Figure 1B)].

### Experimental Design and Soil Sampling

In October of 2019, study sites of ∼0.7 and ∼1 ha in Pumanque and Requínoa, respectively, were chosen for this study. Within these, burned and unburned (undisturbed) areas were selected (Supplementary Figure 2), which represents the main variation source (treatment) in this study. Thus, Pumanque was surveyed 33 months following land burning while Requínoa was evaluated 17 months after fire occurrence. At each burned and unburned areas, three experimental plots of 20 × 20 m were marked (total = 12), and these were separated from the center of each other by 40–60 m, where the closest plots between burned and unburned areas were approximately 10 m apart from their border (Supplementary Figure 2). Inside each experimental plot, a 5 × 5 m grid was projected to obtain nine equidistant points from which soil samples were obtained (Supplementary Figure 3). At each sampling point, a radial buffer area of 1 m was selected and shovel excavated (up to rooting deep, 5–10 cm) to aseptically obtain (1) rhizosphere soil samples by hand shaking and collecting soil attached to roots of all herbaceous species described for each site (Supplementary Figure 1), and (2) bulk soil samples from soil not attached to plant roots. Rhizosphere and bulk soil samples (soil compartments) from each of the nine sampling points per plot were pooled to obtain 12 composite samples for bulk soil (∼2 kg each) and 12 composite samples for rhizosphere (∼300 g each) in total. Thus, within each study location (i.e., Pumanque and Requínoa), the studied variation sources were (1) treatment (burned and unburned soils) and (2) soil compartment (bulk soil and rhizosphere). For every composite soil sample, an aliquot was kept at 4°C for gravimetric water content and physiological profiling, a second portion was stored at −80°C until DNA extraction and further molecular analyses, and the rest was air-dried at room temperature for physicochemical analyses.

### Physicochemical Analysis

Soil physicochemical properties, except for gravimetric water content (GWC), were measured only for bulk soil samples due to the small amount of rhizosphere samples obtained. The GWC was determined by oven-drying 10 g of samples at 105°C for 48 h. Soil pH and electrical conductivity (EC) were determined in a 1:2.5 and a 1:5 (w/v) water extract, respectively (Sadzawka et al., 2006). Nitrate (NO_3_^–^) and ammonium (NH_4_^+^) contents were determined by a distillation-titration method using a 2M potassium chloride (KCl) extraction solution (Sadzawka et al., 2006). Organic matter (OM) was assessed by acid oxidation and colorimetric measurement (Sadzawka et al., 2006). Total carbon (C) and nitrogen (N) contents were determined by the Dumas dry combustion method using a LECO TRUSPEC analyzer (Leco Corporation, MI, United States). Total elements (P, K, Fe, Mg, S, Mo, Na, Ca) were obtained from acid digestion and later determined by inductively coupled plasma atomic emission spectrometry (ICP-AES) (Agilent Technologies, Victoria, Australia) (U.S. EPA, 2007). Soil bulk density (BD) was determined by the clod method (Baver et al., 1973); clay, silt, and sand contents (soil texture) were determined by the soil hydrometer method (Bouyoucos, 1962); and aggregate stability (AS) was determined by wet sieving (Kemper and Rosenau, 1986), using a wet sieving apparatus (Eijkelkamp Soil & Water, Giesbeek, Netherlands).

### Microbial Community-Level Physiological Profile (CLPP)

Carbon source utilization patterns of soil microbial communities, also known as community-level physiological profiles (CLPPs) (Garland and Mills, 1991), were assessed using Biolog EcoPlates (Biolog Inc., Hayward, CA, United States) for all samples (i.e., including both bulk soil and rhizosphere samples). Every plate included the following 31 different carbon sources: (1) carbohydrates: α-D-lactose, i-erythritol, D-xylose, β-methyl-D-glucoside, D-cellobiose, D-mannitol, *N*-acetyl-D-glucosamine; (2) carboxylic acids: 2-hydroxy-benzoic acid, 4-hydroxy-benzoic acid, D-glucosaminic acid, pyruvic acid methyl ester, α-ketobutyric acid, D-galactonic acid γ-lactone, D-malic acid, itaconic acid, γ-hydroxy-butyric acid, D-galacturonic acid; (3) amino acids: L-threonine, L-phenylalanine, glycyl-L-glutamic acid, L-asparagine, L-arginine, L-serine; (4) polymers: α-cyclodextrin, glycogen, tween 80, tween 40; (5) miscellaneous: D,L-α-glycerol-phosphate, glucose-1-phosphate; and (6) amines/amides: phenylethylamine, putrescine. In addition, a blank well was considered in triplicates. Each Biolog EcoPlate well was inoculated with a 150-μl aliquot of a 10^–3^ soil dilution obtained from 5 g of soil suspended in 45 ml of sterile saline solution (0.85% NaCl). The plates were incubated at 25°C and absorbance at 590 nm was determined after 24, 48, 72, 96, 120, 144, and 168 h using the Infinite 200 PRO NanoQuant Microplate Reader (Tecan Group Ltd., Männedorf, Switzerland). At each time interval, optical density (OD) values were corrected by subtracting the control (blank well) values from each plate well. The OD values at 120 h for each C-substrate by sample were averaged to determine microbial catabolic activity, this time corresponded to the mid-exponential growth phase of the average well color development (AWCD-CLPP) (Garland, 1996; Sofo and Ricciuti, 2019).

### Soil DNA Extraction and Molecular Analyses

Soil DNA was isolated from 0.25 g of fresh soil from all samples using the DNeasy PowerSoil DNA isolation kit (QIAGEN, Valencia, CA, United States) following the manufacturer’s instructions. Prokaryotes (bacteria and archaea) were surveyed by quantitative PCR (qPCR) assay and bar-coded amplicon sequencing using the primers 515F (5′ GTG YCA GCM GCC GCG GTA A 3′) (Parada et al., 2016) and 806R (5′ GGA CTA CNV GGG TWT CTA AT 3′) (Apprill et al., 2015) complementary to the V4 region of the 16S rRNA genes. For qPCR, a TaqMan probe (16S probe 5′ TGT AGC RGT GAA ATK CGT AG 3′) was designed for the conserved sequence region amplified by 515F and 806R, by which the dye 6-carboxy-fluorescein (6-FAM) and the Black Hole Quencher (BHQ1) were attached to their 5′ and 3′ ends, respectively. The qPCRs were carried out on a StepOne Plus Real Time PCR System (Thermo Fisher Scientific, United States), in a final volume of 20 μl, containing 10 μl of NZY qPCR Probe Master Mix ROX plus (NZYTech), 0.9 μM of the probe, 0.4 μM of the amplification primers, 2 μl of template DNA, and ultrapure water up to 20 μl. The reaction mixture was incubated as follows: an initial incubation at 95°C for 10 min, followed by 40 cycles of denaturation at 95°C for 15 s, hybridization at 50°C for 1 min, extension at 60°C for 1 min, and a final extension step at 60°C for 30 s. The intensity of the fluorescence emitted by the probe at each cycle of the PCR reaction was registered by using the software StepOne (Applied Biosystems, Foster City, CA, United States), which allowed the estimation of the quantification cycle (Cq). Standard for qPCR was prepared from a known unburned sample (ranging from 5 × 10^10^ to 5 × 10^6^ 16S copy numbers). This standard was obtained from the amplification of the 16S rRNA region complementary to the 515F/806R primer set, which was carried out on a Bio-Rad T100 Thermal Cycler (Bio-Rad, United States), in a final volume of 25 μl, containing 2.5 μl of template DNA, 0.5 μM of the primers, 12.5 μl of Supreme NZYTaq 2 × Green Master Mix (NZTech), and ultrapure water up to 25 μl. The PCR reaction mixture was incubated as follows: an initial denaturation at 95°C for 5 min, followed by 25 cycles of 95°C for 30 s, 50°C for 45 s, 72°C for 45 s, and a final extension step at 72°C for 10 min. The PCR product was purified and used to generate the standard curve in the qPCR experiment. The standard curve was performed by a five-fold dilution series of 16S copy numbers from the qPCR standard by plotting the Cq values against the total 16S rRNA gene copy numbers (Jiang, 2018). Efficiency and *R*^2^ values for the 16S quantification were 84.65 and 0.99%, respectively.

Bar-coded amplicon sequencing was performed on an Illumina MiSeq PE300 platform (Illumina, San Diego, CA, United States) at the AllGenetics & Biology SL laboratory service facility (A Coruña, Spain). PCRs were carried out in a final volume of 25 μl, containing 2.5 μl of template DNA, 0.5 μM of the primers, 12.5 μl of Supreme NZYTaq 2 × Green Master Mix (NZYTech, Lisbon, Portugal), and ultrapure water up to 25 μl. The reaction mixture was incubated as follows: an initial denaturation at 95°C for 5 min, followed by 25 cycles of 95°C for 30 s, 50°C for 45 s, 72°C for 45 s, and a final extension step at 72°C for 10 min. The oligonucleotide indices that are required for multiplexing different libraries in the same sequencing pool were attached in a second PCR round with identical conditions but only 5 cycles and 60°C as the annealing temperature. No template controls were included to check for cross-contamination during library preparation. The libraries were run on 2% agarose gels stained with GreenSafe (NZYTech, Lisbon, Portugal) and imaged under UV light to verify the library size. Libraries were purified using the Mag-Bind RXNPure Plus magnetic beads (Omega Bio-tek, Norcross, GA, United States), following the instructions provided by the manufacturer. Then, they were pooled in equimolar amounts according to the quantification data provided by the Qubit dsDNA HS Assay (Thermo Fisher Scientific, Waltham, MA, United States).

### Processing and Analysis of Sequencing Data

Sequencing data were processed with a customized pipeline largely based on “VSEARCH” (Rognes et al., 2016). Illumina MiSeq reads can be found in the Sequence Read Archive (SRA) of the National Center for Biotechnology Information (NCBI) under accession number PRJNA784510. PhiX reads were removed with Bowtie2 (Langmead and Salzberg, 2012) and primer sequences were trimmed with Cutadapt (Martin, 2011). Paired-end reads were merged by using the *fastq_mergepairs* algorithm in “VSEARCH” and quality filtered using a maximum expected error of one using the *fastq_filter* algorithm in “VSEARCH” (Edgar and Flyvbjerg, 2015). Merged and quality filtered sequences were dereplicated using *derep_fulllength* algorithm in “VSEARCH” and the UNOISE algorithm implemented in “VSEARCH” (Edgar, 2016) was used to retrieve amplicon sequence variants (ASVs). The *uchime* algorithm implemented in “VSEARCH” (Edgar et al., 2011) was applied to detect and remove chimeric ASVs. Remaining ASVs were verified by using Metaxa2 to evaluate the presence of non-target sequences for the 16S rRNA gene (Bengtsson-Palme et al., 2015). The final ASV table was obtained by mapping the high-quality sequences against the verified ASV centroids using the *usearch_global* algorithm implemented in “VSEARCH”. Taxonomic classification was performed by using the Naïve Bayesian classifier *Sintax* implemented in “VSEARCH” (Edgar, 2016) to classify the sequences against the non-redundant SILVA v132 database (Quast et al., 2012). ASVs assigned to mitochondria, chloroplasts, and eukaryotes were removed. Rare ASVs with an abundance < 0.005%, sparse ASVs that occur in less than four samples (which represent 1/6 of the sampling effort), and ASV outliers for which the greatest abundance was more than 0.15 times in a sample compared with the second most abundant value across the other samples were removed from the ASV table. Finally, the filtered ASV table was normalized by iterative subsampling with 100 iterations using the *rrarefy* function implemented in the “*vegan*” package in R (Oksanen et al., 2020).

### Statistical Analysis

Statistical analysis were performed in R statistics version 3.5.1 (R Core Team, 2021). Treatments (burned vs. unburned) and soil compartments (bulk soil and rhizosphere) were considered as the variation sources for statistical analyses, while research location (i.e., sites) was not considered due to the natural differences in biophysical characteristics described previously. The analysis for physicochemical properties of bulk soil samples were performed following normality and homoscedasticity confirmation. To assess differences within treatments (i.e., burned and unburned conditions), a Student’s *t*-test was applied for each of the variables analyzed. For CLPP data sets, a two-way ANOVA considering treatment and soil compartment (i.e., bulk and rhizosphere soil) was performed after normality and homoscedasticity were confirmed. These datasets included the AWCD-CLPP values and the diversity of substrate utilization, determined by the Shannon diversity index (Shannon-CLPP) by: H′ = −Σ pi × ln pi (Shannon, 1948), where pi is the ratio of the respiration rate of every single C-substrate to the sum of all substrates, both obtained from OD values at 120 h of incubation. A heatmap with hierarchical clustering (based on Euclidean distance) was performed for the 31 different carbon sources, considering the average of each C-substrates per treatment and compartment, with the function *heatmap* from the package “stats” (R Core Team, 2021). The rarefied sequencing data were used to determine α-diversity indexes (ASVs, as observed richness, Shannon diversity index, and Pielou’s evenness) with the *diversity* function in “vegan.” A Spearman correlation analysis between soil physicochemical, AWCD-CLPP, Shannon-CLPP, and microbial α-diversity indexes was applied with the function *cor* from the package “corrplot” (Wei and Simko, 2021). To assess differences within treatments and soil compartment for each of these indexes, a two-way ANOVA was conducted after normality and homoscedasticity were confirmed. Differences in β-diversity were assessed by calculating Bray–Curtis dissimilarities based on the iteratively rarefied ASV abundance tables with a PERMANOVA and PERMDISP by using the functions *adonis2* and *betadisper* from the package “vegan”, respectively. This allowed the detection of factors with significant effects on prokaryotic community structure. Thus, a canonical analysis of principal coordinates (CAP, Anderson and Willis, 2003) was applied with the function *CAPdiscrim* from the package “BiodiversityR” (Kindt and Coe, 2005). A distance-based redundancy analysis (dbRDA, Legendre and Andersson, 1999) was used to constrain β-diversity patterns by the bulk soil physicochemical properties using the function *dbrda* and *envfit* from the package “vegan.” In addition, relative abundance data of ASVs was Z-transformed to show relative change between burned and unburned soils. A univariate PERMANOVA (Anderson, 2001) and PERMDISP (Anderson, 2001) were applied on Z-transformed relative abundance of ASVs at the phylum level and also at the order taxa and genus taxa levels to evaluate treatment effects on each soil compartment with the *adonis2* and *betadisper* function from the package “vegan” (Oksanen et al., 2020).

## Results

### Soil Abiotic Properties

Statistically significant differences between burned and unburned bulk soils were observed for NH_4_^+^ and NO_3_^–^ in Pumanque (taken 33 months after fire occurrence) and for P, Fe, Na, and AS in Requínoa (taken 17 months after fire occurrence) (Supplementary Table 1). In Pumanque, NH_4_^+^ contents almost doubled in burned soils, and NO_3_^–^ contents decreased by approximately two thirds compared with unburned soils (Supplementary Table 1). In Requínoa, total P and Fe contents dropped by 43 and 14% in burned soils, respectively, and Na increased by almost 70% in burned soils. In terms of physical properties, AS was significantly lower in burned soils in Requínoa. Although non-significant, NH_4_^+^ and NO_3_^–^ contents showed opposite results in Requínoa compared with Pumanque. In addition, also not significant, a decreasing trend in OM, C, and N and an increasing trend in soil pH and BD were observed for burned soils at both sites.

### Microbial Community-Level Physiological Profile (CLPP)

**FIGURE 2:**
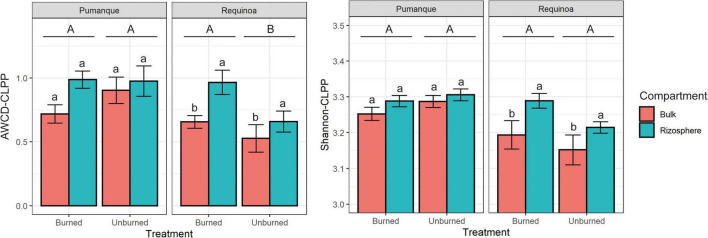
Bar plots of the average well color development (AWCD) (mean ± SE, *n* = 3) and Shannon diversity index based on CLPP (Shannon-CLPP) (mean ± SE, *n* = 3) of microbial communities for each study location contrasting treatments (burned vs. unburned) and soil compartment (bulk soil vs. rhizosphere soil). Different uppercase letters show significant (*p* < 0.05) differences by treatment. Different lowercase letters show significant (*p* < 0.05) differences by compartment within each treatment. Interactions between treatment and compartment were not significant (*p* > 0.05).

Microbial catabolic activity and diversity of substrate utilization [here assessed by AWCD-CLPP and Shannon diversity index (Shannon-CLPP), respectively] responded differently to forest fires at both sites. In Pumanque (assessed 33 months after fire), there were no effects of burning on AWCD-CLPP or Shannon-CLPP (Table 1). In addition, no differences in carbon utilization were observed between bulk and rhizosphere samples at this site (Figure 2), although higher values for AWCD and Shannon were observed for rhizosphere samples in both burned and unburned conditions (Figure 2). On the contrary, in Requínoa (assessed burned 17 months after fire), soils showed significantly higher catabolic activity, with AWCD values in the burned soils being 37% higher than in the unburned soils (Table 1 and Figure 2). Significant differences were also observed between soil compartment (Table 1), with rhizosphere samples showing higher AWCD values than bulk soil samples in both burned and unburned conditions (Figure 2). Shannon diversity of substrate utilization was also significantly higher in the rhizosphere when compared with the bulk soil, whereas burning had no effect (Table 1 and Figure 2).

**TABLE 1:** *F*-values from two-way ANOVA for biological activity and α-diversity of prokaryotic communities, permutational multivariate analysis (PERMANOVA), and permutation test for homogeneity of multivariate dispersion (PERMDISP) for prokaryotic community (β-diversity) (the table is split by study locations).

Pumanque	AWCD-CLPP	Shannon-CLPP	16S rRNA copy number (*log*)	Richness_16S	Shannon_16S	Pielou’s Evenness_16S	β-Diversity_16S PERMANOVA	β-Diversity_16S PERMDISP
Treatment	0.887	2.354	7.635 *	3.652	35.148***	62.87***	7.95***	16.98**
Source	3.387	2.539	2.982	0.425	8.564*	17.30**	5.51***	4.61
Treatment:source	1.144	0.264	0.913	1.037	15.309**	29.61***	1.59	1.19

**Requínoa**	**AWCD-CLPP**	**Shannon-CLPP**	**16S rRNA copy number (*log*)**	**Richness_16S**	**Shannon_16S**	**Pielou’s Evenness_16S**	**β-Diversity PERMANOVA**	**β-Diversity PERMDISP**

Treatment	6.361*	3.39	11.240*	8.331*	13.694**	14.573**	6.43**	0.17
Source	6.511*	6.243*	1.514	0.000	0.380	0.092	0.62	0.03
Treatment:source	1.053	0.271	2.007	0.119	0.000	0.051	0.49	0.04

Treatment = Burned vs. unburned. Source = bulk soil vs. rhizosphere. Biological indices followed by “CLPP” were obtained with data from Biolog EcoPlates sequencing. Biological indices followed by “16S” were obtained with data from Illumina sequencing. log = logarithmic transformed variables. AWCD-CLPP = average well color development. Significance codes are based on p-values as follows: “***” 0.001, “**” 0.01, “*” 0.05.

**FIGURE 3:**
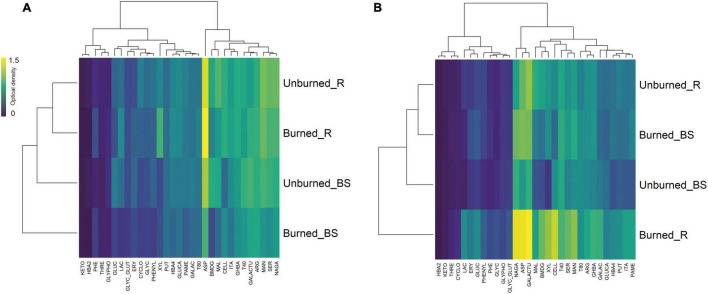
Clustered heatmaps based on the average (*n* = 3) of carbon source utilization patterns of soil microbial communities, also referred as community-level physiological profile (CLPP) for **(A)** Pumanque and **(B)** Requínoa, by treatment (burned vs. unburned) and soil compartment (R = rhizosphere; BS = bulk soil). Row dendrograms show the Euclidean distance between treatments, while column dendrograms show the distance between the utilization of C sources. Yellow boxes represent higher microbial C utilization. The following 31 different carbon sources were used: (1) carbohydrates: α-D-lactose (LAC), i-erythritol (ERY), D-xylose (XYL), β-methyl-D-glucoside (BMDG), D-cellobiose (CELL), D-mannitol (MAN), *N*-acetyl-D-glucosamine (NAGA); (2) carboxylic acids: 2-hydroxy-benzoic acid (2HBA), 4-hydroxy-benzoic acid (4HBA), D-glucosaminic acid (GLUCA), pyruvic acid methyl ester (PAME), α-keto-butyric acid (KETO), D-galactonic acid γ-lactone (GALAC), D-malic acid (MAL), itaconic acid (ITA), γ-hydroxy-butyric acid (GHBA), D-galacturonic acid (GALACTU); (3) amino acids: L-threonine (THRE), L-phenylalanine (PHE), glycyl-L-glutamic acid (GLYC), L-asparagine (ASP), L-arginine (ARG), L-serine (SER); (4) polymers: α-cyclodextrin (CYCLO), glycogen (GLYC), Tween 80 (T80), Tween 40 (T40); (5) miscellaneous: D,L-α-glycerol-phosphate (GLYPHO), glucose-1-phosphate (GLUC); and (6) amines/amides: phenylethylamine (PHE), putrescine (PUT).

Clustered heatmaps for the average (*n* = 3) of each C-substrate utilization showed different catabolic capacity for soil microbial communities in both study locations (Figure 3), where utilization decreased for recalcitrant substrates (e.g., polymeric compounds and carboxylic acids) compared with labile C sources (e.g., carbohydrates). Soil microbial communities from Requínoa showed higher C utilization capacity than those from Pumanque, with unclear trends regarding the type of C, while C sources in the form of carbohydrates and carboxylic acids were generally more used by soil microorganisms in Pumanque. A closed look to C source consumption indicated that L-asparagine (ASP) was the C source most consumed in soils from Pumanque, especially rhizosphere of burned soils. Similarly, ASP was the C source most consumed in soils from Requínoa particularly in rhizosphere of burned soils, in addition to galacturonic acid (GALACTU) and *N*-acetyl-D-glucosamine (NAGA), which were specific for this site.

### Prokaryotic Abundance, Alpha and Beta Diversity, and Taxonomy Assignments

**FIGURE 4:**
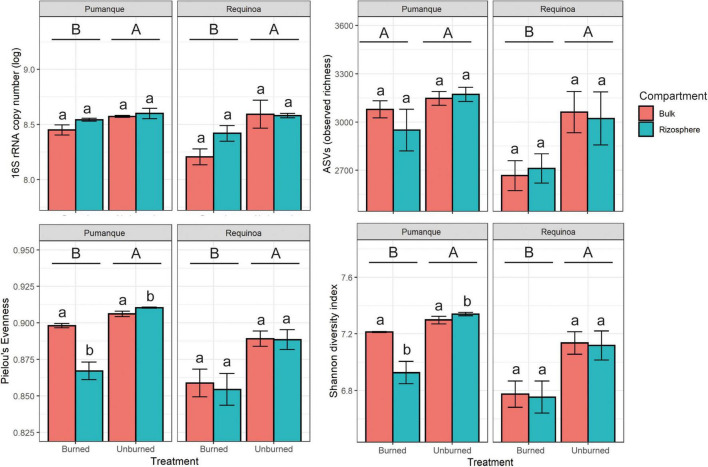
Bar plots of abundance (mean ± SE, *n* = 3) and alpha diversity (mean ± SE, *n* = 3) of prokaryotic communities for each study locations showing effect of treatment (burned vs. unburned) and soil compartment (bulk soil vs. rhizosphere soil). Different uppercase letters show significant (*p* < 0.05) differences by treatment. Different lowercase letters show significant (*p* < 0.05) differences by compartment. Interactions between treatment and compartment were not significant (*p* < 0.05).

DNA-based analyses were more sensitive to detect the effects of fire than the substrate utilization profiles (Table 1). Unburned soils in both Pumanque and Requínoa showed significantly higher 16S rRNA gene copy numbers when compared with the burned soils (Table 1 and Figure 4). No differences in gene copy numbers were observed between bulk and rhizosphere soils (Table 1 and Figure 4).

A total of 3,782 curated prokaryote ASVs were obtained across all samples. Statistically significant differences in observed richness were detected only between burned and unburned samples in Requínoa (Table 1), where ASVs in burned soils decreased by 12% (2,687 ± 143.7) when compared with unburned soils (3,041 ± 229.1) (Figure 4). No differences in richness were observed between soil compartments. Shannon diversity (Shannon-16S) and Pielou’s evenness (Evenness-16S) were higher in unburned soils at both study locations, with alterations due to soil compartment only in Pumanque (Table 1 and Figure 4).

**FIGURE 5:**
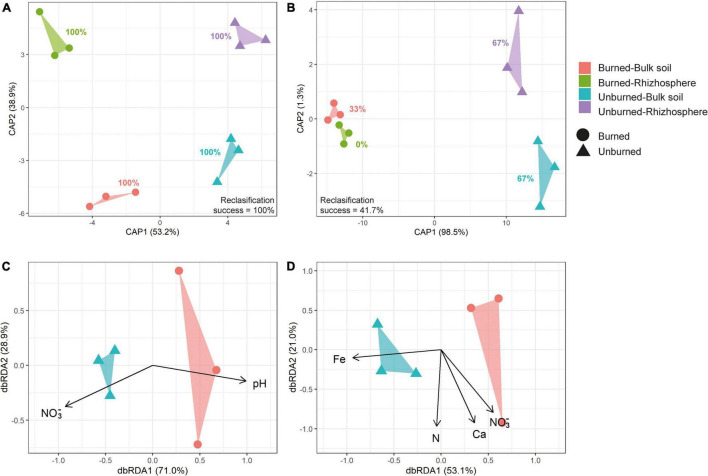
Beta diversity for prokaryotic communities from burned and unburned bulk and rhizosphere soil samples. Canonical analysis of principal coordinates (CAP) based on Bray–Curtis dissimilarities constrained by burned and unburned as well as bulk and rhizosphere soils from Pumanque **(A)** and Requínoa **(B)**. The CAP reclassification (in percent) for each treatment is shown next to each cluster. Distance-based redundancy analysis (dbRDA) based on Bray–Curtis dissimilarities constrained by soil physicochemical properties (pH, NO_3_^–^, and total contents of Fe, N, and Ca) in burned and unburned bulk soils from Pumanque **(C)** and Requínoa **(D)**. Soil physicochemical properties (explanatory variables) are shown by solid eigenvectors. PERMANOVA was performed by each site independently (Table 1).

Beta diversity analyses showed differences in soil prokaryotic community structure according to fire occurrence at both study locations (Table 1 and Figure 5, and Supplementary Figure 6). Moreover, in Pumanque, communities also separated apart according to their compartment (bulk soil and rhizosphere), and larger dissimilarities were observed between rhizosphere of burned and unburned soils when compared with the bulk soils (Figure 5A). In Requínoa, prokaryotic community structures also showed dissimilarities between burned and unburned soils (Figure 5B), while differences between compartment were not conclusive due to low CAP reclassification success. On the other hand, Bray–Curtis distances between burned and unburned soils from Requínoa were approximately two times greater than Pumanque. Distance-based redundancy analysis (dbRDA) for bulk soil data sets showed discrimination of prokaryote communities by forest fire associated to some soil physicochemical properties. The most parsimonious and significant dbRDA models showed that soil pH and NO_3_^–^ content explained prokaryotic community changes in Pumanque (Figure 5C), while contents of NO_3_^–^, and total contents of Fe, N, Ca, and NO_3_^–^ were important explanatory variables of prokaryote community changes in Requínoa (Figure 5D).

**FIGURE 6:**
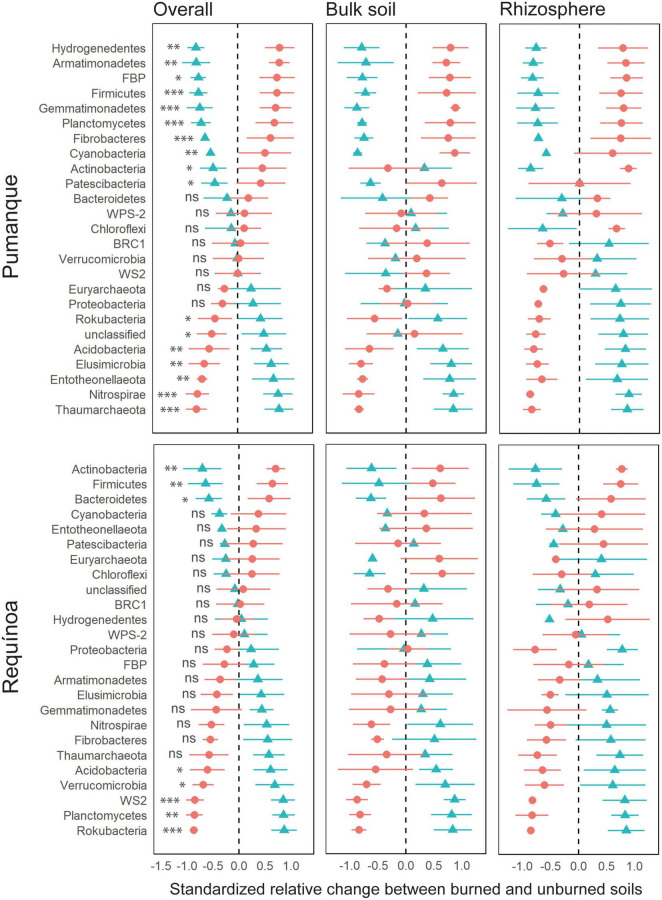
Relative change (z-transformed) of prokaryote abundance, at the phylum level, between unburned (blue triangle) and burned (red spot) soils for Pumanque and Requínoa. The panel to the left shows the relative change in abundance from the overall mean ± SE (considering bulk soil and rhizosphere). The middle and right panels show the relative change in abundance for bulk and rhizosphere soils, respectively. Bulk soil and rhizosphere panels show the relative change of phylum between burned and unburned soil for each soil compartments. The significance of the PERMANOVA test is shown at left side of each relative change in the overall panel. Significance codes are based on *p*-values as follows: “***” 0.001, “**” 0.01, “*” 0.05, ns = not significant. PERMANOVA was not applied on bulk soil and rhizosphere due to scarce data.

Taxonomy assignments resulted in a total of twenty-two and two classified bacterial and archaeal phyla, respectively. The most abundant phyla (>70%) across samples were Proteobacteria, Actinobacteria, and Acidobacteria (Supplementary Figure 5A). A total of sixteen and eight classified prokaryotic phyla were affected by fire occurrence in Pumanque and Requínoa, respectively. In Pumanque, forest fire negatively affected the relative abundance of Thaumarchaeota, Nitrospirae, Entotheonellaeota, Elusimicrobia, Acidobacteria, and Rokubacteria in both soil compartments, except for Rokubacteria with differences in rhizosphere samples only (Figure 6). Contrarily, relative abundance of the candidate division FBP, Gemmatimonadetes, Planctomycetes, Fibrobacteres, Hydrogenedentes, Armatimonadetes, Cyanobacteria, Firmicutes, Actinobacteria, and Patescibacteria increased in burned soils as compared with unburned soils, with similar responses in bulk soil and rhizosphere. However, relative abundance of Actinobacteria and Patescibacteria only increased in rhizosphere and bulk soil samples, respectively. In Requínoa, forest fire negatively affected the relative abundance of Rokubacteria, WS2, Planctomycetes, Acidobacteria, and Verrucomicrobia, which showed similar responses in bulk soil and rhizosphere. In contrast, Actinobacteria, Firmicutes, and Bacteroidetes increased in burned soils, with similar responses in both soil compartments.

From the classified phyla significantly affected by fires, there were also 33 and 26 orders showing significant changes due to fires in Pumanque and Requínoa, respectively (Supplementary Figure 7). In Pumanque, the relative abundance of 16 orders decreased due to fire occurrence, which mainly belonged to Actinobacteria (Supplementary Figure 7). Nitrososphaerales (Thaumarchaeota) was also among the taxa most affected by fire at this site, in both soil compartments. Instead, 17 orders increased in burned soils from Pumanque, which mainly belonged to Actinobacteria, Gemmatimonadetes, Planctomycetes, and Acidobacteria (Supplementary Figure 7). Among them, Hydrogenedentiales (Hydrogenedentes) showed the greatest increase in relative abundance due to fire occurrence. In Requínoa, 17 orders were negatively affected by fire and most of them belonged to Actinobacteria, Acidobacteria, and Planctomycetes (Supplementary Figure 7). Only ten orders increased in relative abundance after fire, which mainly belonged to Actinobacteria (Supplementary Figure 7). Fires reduced the relative abundance of Pyrinomonadales (Acidobacteria), Rokubacteriales (Rokubacteria), Solirubrobacterales (Actinobacteria), and Pseudonocardiales (Actinobacteria) at both locations. Contrarily, Bacillales (Firmicutes) was the only order that consistently increased in burned soils at both study locations (Supplementary Figure 7).

## Discussion

Soil physicochemical properties did not show strong differences according to fire occurrence, especially in Pumanque where only clear alterations of NO_3_^–^ and NH_4_^+^ were found in burned soils. In fire-affected soils, NH_4_^+^ is known to form as a result of organic matter mineralization induced by land burning, which is later transformed to NO_3_^–^ by nitrification (Certini, 2005). However, this latter transformation could be differently altered after fire, as some findings have reported a decrease in nitrifying microorganisms after fires (Acea and Carballas, 1996), while others have reported higher nitrification rates occurring 12 months after fire (Covington and Sackett, 1992). Thus, lower nitrification rates in Pumanque, and the opposite trend for Requínoa, could partly explain differences in NO_3_^–^ and NH_4_^+^ after forest fire, which is similar to those reported by Ball et al. (2010) who found higher nitrification rates in recent burned soil (12 years old) compared with a site without fire for 75 years. In addition, NH_4_^+^ and NO_3_^–^ contents also respond to their different mobility in soils, where NO_3_^–^ is prone to leaching over time while NH_4_^+^ could be retained in burned soils due to adsorption to organic matter (OM) or mineral surfaces (Mroz et al., 1980). A combination of these biotic and abiotic processes would likely relate to the negative correlations observed between NO_3_^–^ and NH_4_^+^ contents in soils from Pumanque and Requínoa, where in the former NH_4_^+^ increased and NO_3_^–^ decreased 33 months after land burning, and in the latter NH_4_^+^ decreased and NO_3_^–^ increased after 17 months of forest fire occurrence. On the other hand, lower P and Fe contents in burned soils from Requínoa might be related to losses of Fe/P bound to organic matter (OM) in fire-affected soils (Norouzi and Ramezanpour, 2013), which might have migrated from surface to lower horizons (Mosugu et al., 1999). In addition, both study locations showed a slight increase in pH in burned soils, which was expected due to the OH^–^ and cation release from the denaturation of OM occurring after fires (Certini, 2005; Alcañiz et al., 2018).

In burned soils, microbial activity can be promoted by the combustion of plant residues that increase C and N input to microorganisms (Díaz-Raviña et al., 1996; Liu et al., 2000), especially in the rhizosphere that is considered as a hotspot for microbial activity in soils (Prashar et al., 2014). Thus, negative effects of forest fires on plant cover and diversity indirectly affect soil microorganisms associated with roots (Certini, 2005; Hart et al., 2005). In our study, forest fires differently affected microbial catabolic activity assessed by Biolog EcoPlates. Influence of land burning was mostly evident in Requínoa, where forest fires increased the microbial catabolic activity, especially in the rhizosphere. Different responses of microbial metabolic fingerprints based on Biolog EcoPlates have been related to vegetation coverage after fires in Mediterranean pine forest (Moya et al., 2021). For instance, microbial catabolic activity has been shown to increase a few months after forest fires, especially under soils with high vegetation cover, while C substrate utilization patterns have also been observed to increase in burned soils, but with no relation to vegetation (Moya et al., 2021). However, opposite results have been reported in desert grassland ecosystems, where fires were shown to reduce soil microbial substrate utilization with variations observed across seasons (Liu et al., 2000). In our study, although herbaceous species richness in Pumanque (assessed 33 months after land burning) was greater than in Requínoa (assessed after 17 months following fires), the latter showed significant increase of substrate usage in burned soils. Thus, conditions in burned soils in Pumanque might closely resemble biological functionality of unburned soils at this site. Despite dissimilar responses of substrate utilization observed in soils from Pumanque and Requínoa after land burning, values were always higher for rhizosphere samples at both sites, supporting the influence of vegetation on this biological indicator (Zhang et al., 2020). Higher values associated to the rhizosphere using CLPP approaches (assessed either by Biolog EcoPlates or MicroResp) in other Mediterranean regions (de Armas-Ricard et al., 2016), and different ecosystems such as boreal coniferous forests (Hernesmaa et al., 2005) or agricultural fields (Sneha et al., 2021), have been attributed to root exudates that serve as C sources for microbial communities (Grayston et al., 1997; Rodríguez-Loinaz et al., 2008).

Carbon substrate utilization capacity has been shown to differ following land burning (Barreiro et al., 2015; Moya et al., 2021). Immediately after experimental fires, C sources in the form of carboxylic acids, amino acids, carbohydrates, and phenolic acids have been shown to greatly increase, while over a year all C source utilization tends to resemble unburned conditions (Barreiro et al., 2015). In our study, no clear patterns were observed for soils from Requínoa; however, soils from Pumanque showed higher utilization of carbohydrates and carboxylic acids, especially in rhizosphere samples of the burned soils. This can be likely due to the recovery of C labile forms reported to occur over a year after fire occurrence (Martín et al., 2009), which in the case of Pumanque could have been promoted by the recovery of herbaceous vegetation. The microbial L-asparagine (ASP) utilization increased in rhizosphere samples in burned soils from Pumanque and Requínoa (as well as other carbohydrates and organic acids in the case of the latter), which can be associated with the release of ammonia by microbial activity of asparaginases (Kandeler et al., 2011). However, as previously mentioned, NH_4_^+^ and NO_3_^–^ contents showed opposite results at each site, which can likely relate to transformation of ammonia to nitrate differently affected at both sites. In the case of Pumanque, the consumption of ASP followed by low nitrification rates, as observed in other ecosystems few years after fire (Cobo-Díaz et al., 2015), can relate to higher ammonia detected at this site, while in Requínoa, ASP utilization followed by rapid microbial uptake and conversion to nitrate (Covington and Sackett, 1992; Hart et al., 2005; Switzer et al., 2012) likely relates to higher nitrate contents registered at this site. These findings support the hypothesis proposed by Yang et al. (2020), who stated that forest fires could stimulate functional microbial groups related to N cycling such as *ureC*, *amoA* (Ball et al., 2010), and *nifH* (Sun et al., 2016) genes.

In our study, the assessments of soil prokaryotic communities, based on molecular ribosomal markers, evidenced a greater sensitivity of this method as compared with CLPP to elucidate changes in response to the occurrence of fires. This might reflect different sensitivity of soil genetic and functional diversity after forest fires under the studied conditions. Although metabolic activity and the diversity of C substrate utilization were not affected by forest fires in Pumanque, burned soils at this site showed lower prokaryote abundance and α-diversity indexes, and clear dissimilarities in community composition between burned and unburned samples, as well as rhizosphere and bulk soils. These findings suggest the presence of functionally redundant microbial groups at this site, which coincides with other studies showing recovery of soil functionality but not bacterial community structure after fires (Pérez-Valera et al., 2019). Similarly, burned soils from Requínoa, which showed higher metabolic activity compared with unburned soils, had lower prokaryote 16S rRNA gene copy numbers and diversity, and clear distinct community structure. These results agree with several studies, including those performed in other Mediterranean regions, that have reported decreases in soil microbial abundance (e.g., direct counts, qPCR) and diversity (e.g., based on DNA data) due to negative effect of heating (Mataix-Solera et al., 2009; Alcañiz et al., 2018; Pressler et al., 2019; Marín and Rojas, 2020). Low prokaryote abundance and diversity in burned soils can be likely associated with initial detrimental effects of forest fires on soil microorganisms followed by slow recovery of genomic pools, which can be partly explained by persistent changes in soil physicochemical properties mentioned previously. Long-term effects of forest fires on soil microbial communities can also be associated with post-fire climate conditions, substrate availability, plant recolonization, changes in soil physicochemical properties, and time after fires, among others (Hart et al., 2005; Mataix-Solera et al., 2009; Switzer et al., 2012; Singh et al., 2017; Li et al., 2019). Based on our results, low 16S rRNA gene copy numbers and α-diversity in burned soils still evidence the legacy of fires despite the time elapsed after fires. This observation supports findings from other ecosystems across the globe, which suggest recovery of soil microbial communities beyond 10 years following fire occurrence (Xiang et al., 2014; Pressler et al., 2019), as opposed to those reporting conditions comparable with unburned counterpart close to 3 years after fires (Bárcenas-Moreno et al., 2011; Sadeghifar et al., 2020). In the case of our study, similar to Mediterranean ecosystems located at other latitudes, legacy of fires in soil microbial community structure and microbial activity is still detectable within an approximate 1- to 3-year period, which is particularly critical in the Chilean ecosystem with no fire-prone vegetation (Rundel et al., 2018, 2016).

Prokaryote communities were dissimilar according to fire occurrence at both study locations. Dissimilarities of prokaryote communities in Pumanque were also observed between bulk and rhizosphere soil samples. Thus, such differences probably resulted from greater vegetation richness that increased the input of organic matter with different quality to bulk soil in addition to C input from rhizosphere, by which these differences were not attributed to forest fires. On the other hand, greater distances between microbial community structures were found between burned and unburned soils from Requínoa compared with Pumanque, which might reflect the greater number prokaryotic genera that significantly changed after fire. In Pumanque, pH and the available NO_3_^–^ content significantly explained variability in the community structure, while available NO_3_^–^ and total Fe, N, and Ca contents were associated with prokaryote community changes in Requínoa. Soil pH and N has been shown to be important factors to determine the prokaryote community composition (Mandakovic et al., 2018; Li et al., 2019; Yang et al., 2020), which we confirm in this study. For instance, Li et al. (2019) reported that soil pH, total N, and available NH_4_^+^ contents were the main drivers of changes in microbial communities affected by fires. Similar to our study, Pérez-Valera et al. (2020) reported that soil nitrogen content and pH were strong predictors of soil microbial functions after forest fire in a Mediterranean region in Spain. Effects of soil pH on bacterial communities can be associated with (1) physiological pressure on soil bacteria, reducing the growth of some taxa unable to survive in some pH ranges, and (2) pH effects on soil properties, such as substrate availability (Mandakovic et al., 2018), which may drive changes in bacterial community composition (Lauber et al., 2009). On the other hand, NO_3_^–^ could be associated with biological functioning described previously since it is immediately leached if not taken up (Li et al., 2019). This also suggests differences in composition of prokaryotic groups involved in N cycling. In this sense, decreasing total Fe content in burned soils from Requínoa might also result in lower abundance of prokaryotes able to synthesize Fe-dependent nitrogenases (Raymond et al., 2004).

In our study, some prokaryote taxa were consistently affected by fires at both sites, likely due to different sensitivity to forest fires reported for several microbial groups (Mataix-Solera et al., 2009). Firmicutes and Actinobacteria significantly increased in burned soils from both locations as also reported by Cobo-Díaz et al. (2015) in a fire-affected forest of the Mediterranean Basin, while Acidobacteria and Rokubacteria were reduced by fires at both research sites. Indeed, the former two phyla have been found to increase following fires due to their resistance to heat (Pérez-Valera et al., 2019). Moreover, Rodríguez et al. (2018) reported an increase in Firmicutes and a decrease in Acidobacteria in Spanish Mediterranean burned soils after 2 and 3 years of fire. Similarly, Li et al. (2019) showed higher abundance of Proteobacteria and Actinobacteria, and lower abundance of Acidobacteria, Verrucomicrobia, and Chloroflexi after wildfires. In our study, Firmicutes found to increase in burned soils at both locations belonged to the order of Bacillales, particularly to the genera *Tumebacillus* and *Bacillus* in the case of Pumanque (Supplementary Figure 9) and to *Tumebacillus*, *Conhella*, and *Paenibacillus* in the case of Requínoa (Supplementary Figure 9). Spore- or endospore-forming bacteria within all these genera have been previously described (Khianngam et al., 2010; Kim and Kim, 2016; Puri et al., 2016). This might explain the increase of relative abundance of Firmicutes in burned soils in our study, as such resistance structures allow to better cope with higher temperature and also promote proliferation of spore- or endospore-forming microbial groups due to spore germination enhanced by fire (Dworkin, 2006; Pérez-Valera et al., 2019). In the case of members of the Acidobacteria, which decreased in burned soils from Pumanque and Requínoa, these belonged to the Pyrinomonadales and DS-100 orders in Pumanque and to the Pyrinomonadales, Acidobacteriales, and Blastocatellales and 11–24 orders in Requínoa. Previous studies have shown that different groups of Acidobacteria have variable responses to changes in soil pH, nutritional conditions, and C source availability (Eichorst et al., 2011; Kielak et al., 2016). It has been shown that Acidobacteria subdivisions 1, 2, 3, 12, 13, and 15 negatively correlate with soil pH, while subdivisions 4, 6, 7, 10, 11, 11, 16, 17, 18, 22, and 25 show an opposite behavior (Kishimoto et al., 1991; Janssen, 2006; Sait et al., 2006; Jones et al., 2009; Kielak et al., 2016); even subdivision 6 can either increase or decrease according to soil pH (Kielak et al., 2016). Thus, increases in soil pH registered in our study as a result of land burning can likely explain the decrease of Acidobacteria susceptible to this condition (Li et al., 2020; Sun et al., 2021). Interestingly, Thaumarchaeota was the archaeal phylum most negatively affected by forest fires in Pumanque, particularly of the order Nitrososphaerales (Supplementary Figure 8). Species of this order can oxidize ammonia (Tourna et al., 2011; Brochier-Armanet et al., 2012; Bustamante et al., 2012), similar to species of Nitrospirae phylum such as those within the Nitrospirales order (Vijayan et al., 2021), which also decreased in abundance after forest fire in Pumanque. This in part might be related to potentially lower rates of nitrification in burned soils from Pumanque.

Our results indicate that forest fires have differently altered prokaryote community-level physiological profiles and community structure in the study sites located in the Mediterranean climate zone of central Chile, providing local evidence that support and complement previous findings in other Mediterranean ecosystems across the globe. Legacy of land burning was still clearly shaping soil prokaryotic community structure rather than community-level physiological profiles, particularly in the most recent fire-affected site. However, we acknowledge that these findings cannot be completely disentangled from site effects (e.g., soil type, vegetation, and climatic conditions); thus, resilience of soil prokaryotic communities can also be explained by these factors. Nonetheless, changes in the relative abundance of coinciding taxonomic groups were observed in fire-affected soils at both locations, despite environmental difference between sites. Moreover, variability between community structures was moderated by changes in soil physicochemical properties known to be affected by fires and possibly by post-fire substrate availability. In an ecosystem that is more frequently affected by fires and whose vegetation is not well adapted to such events, as is the case for Mediterranean ecosystems of central Chile, our results allow for a better understanding of the state of soil prokaryotes and their physiological conditions after different time periods following fire, and suggest that biotic belowground responses to fire occurrence at this particular Mediterranean ecosystem are comparable with other Mediterranean biomes across the globe. These findings highlight the importance of including such soil microbiological assessments in combination to aboveground conditions to better understand forest ecosystem resilience and restoration processes under local conditions where natural recovery is hampered.

## Data Availability Statement

The dataset generated for this study can be found in the raw sequencing data deposited in the Sequence Read Archive (SRA) of NCBI under accession number PRJNA784510.

## Author Contributions

HA, CY, and CR designed the research and performed the laboratory work. HA, CY, TG-C, and MH performed the data analysis. HA and CR wrote the manuscript and designed the tables and figures. CY, TG-C, and MH made revisions of the manuscript. All authors revised the article and approved the final version.

## Conflict of Interest

The authors declare that the research was conducted in the absence of any commercial or financial relationships that could be construed as a potential conflict of interest.

## Publisher’s Note

All claims expressed in this article are solely those of the authors and do not necessarily represent those of their affiliated organizations, or those of the publisher, the editors and the reviewers. Any product that may be evaluated in this article, or claim that may be made by its manufacturer, is not guaranteed or endorsed by the publisher.
