# Supplementary material for: Microbial Community-Level Physiological Profiles and Genetic Prokaryotic Structure of Burned Soils Under Mediterranean Sclerophyll Forests in Central Chile
Source: Front Microbiol. 2022 Apr 28;13:824813. doi: 10.3389/fmicb.2022.824813 (PMC9096493; doi:10.3389/fmicb.2022.824813)
Supplement: Supplementary file 1 [file Data_Sheet_1.docx]

Supplementary Material

# Supplementary Figures and Tables

## Supplementary Figures


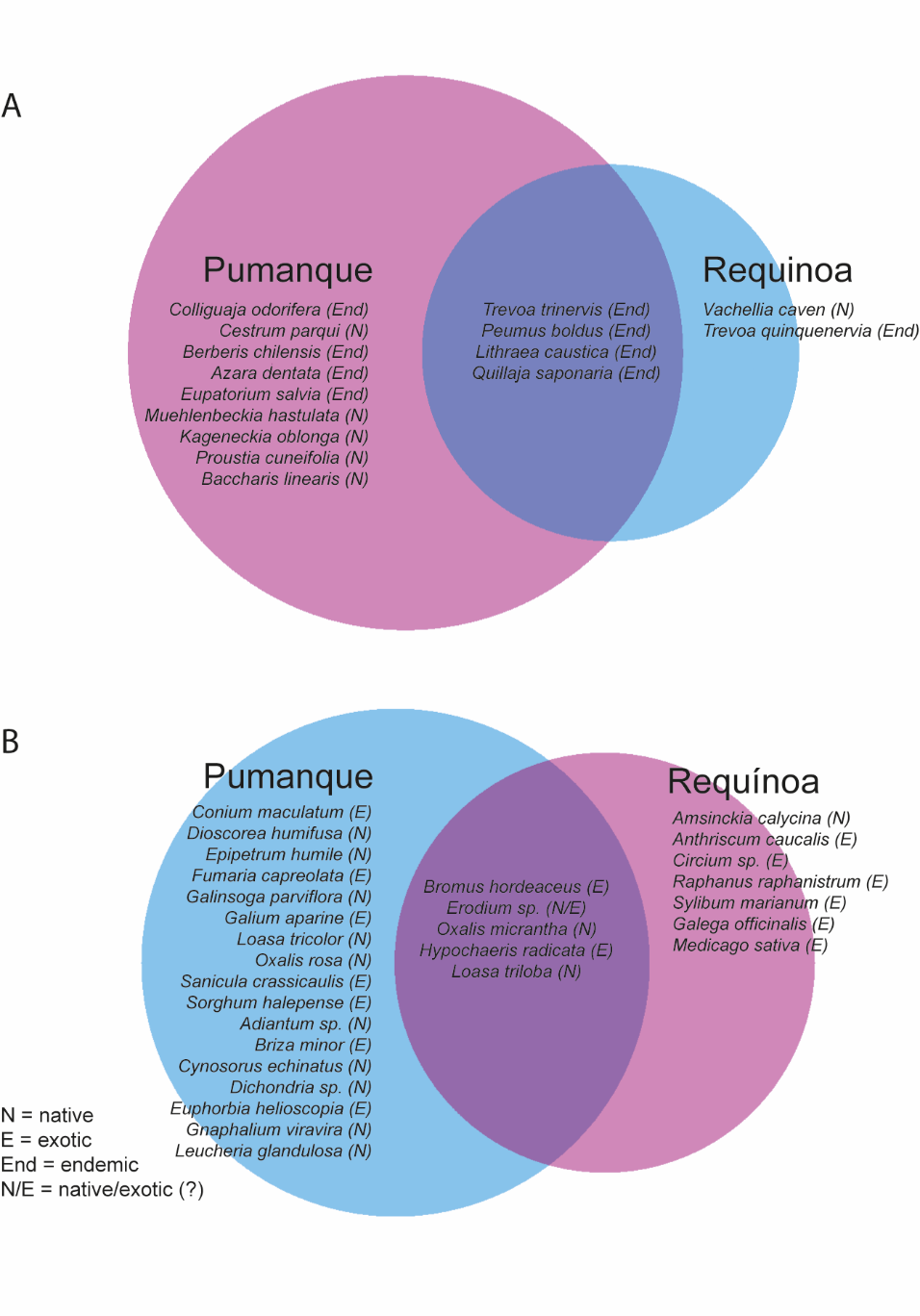


**Supplementary Figure 1.** Venn diagram of woody (A) and herbaceous (B) species present at each study locations in October of 2019.


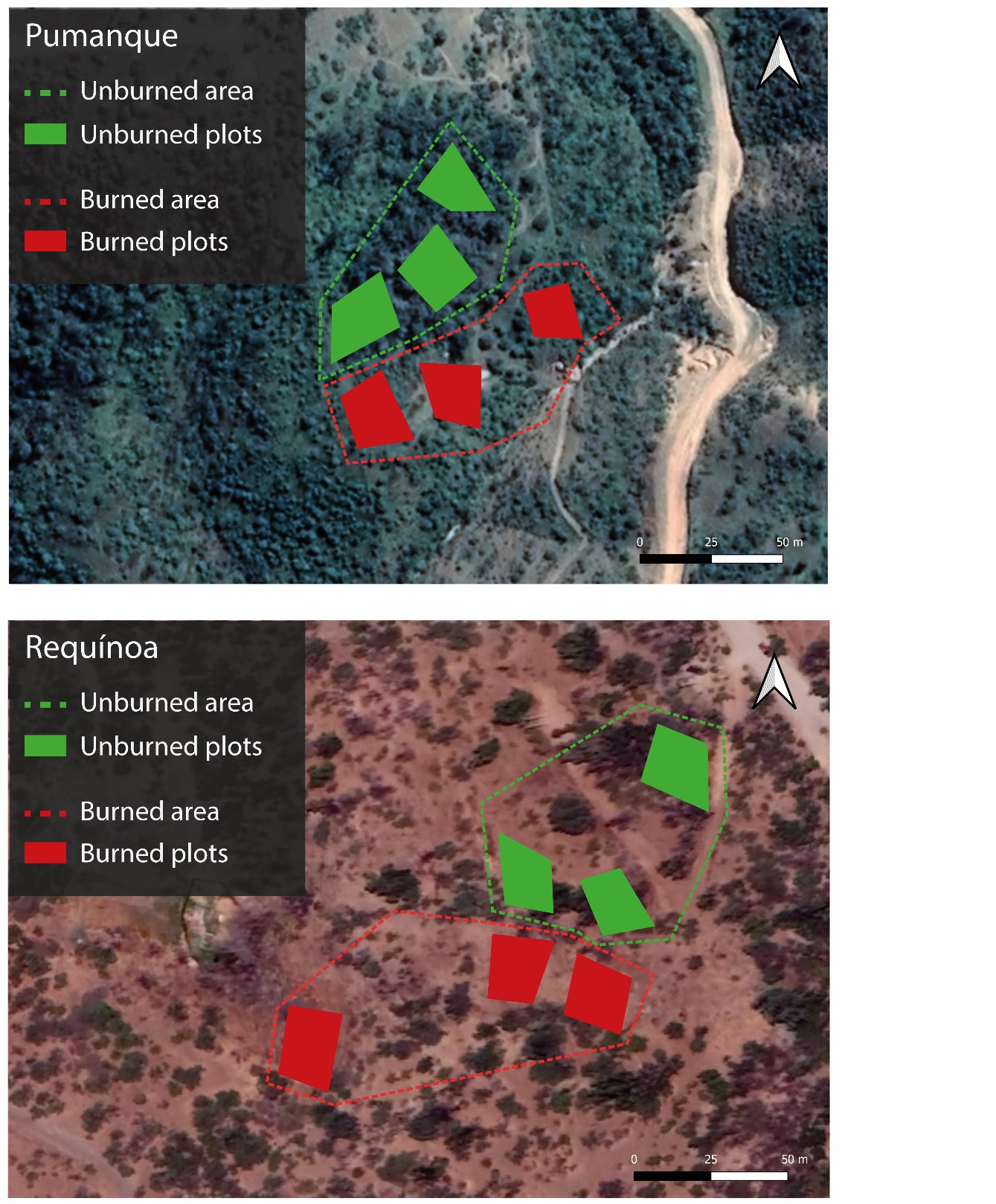


**Supplementary Figure 2**. Distribution of experimental burned and unburned areas and plots in Pumanque and Requínoa.


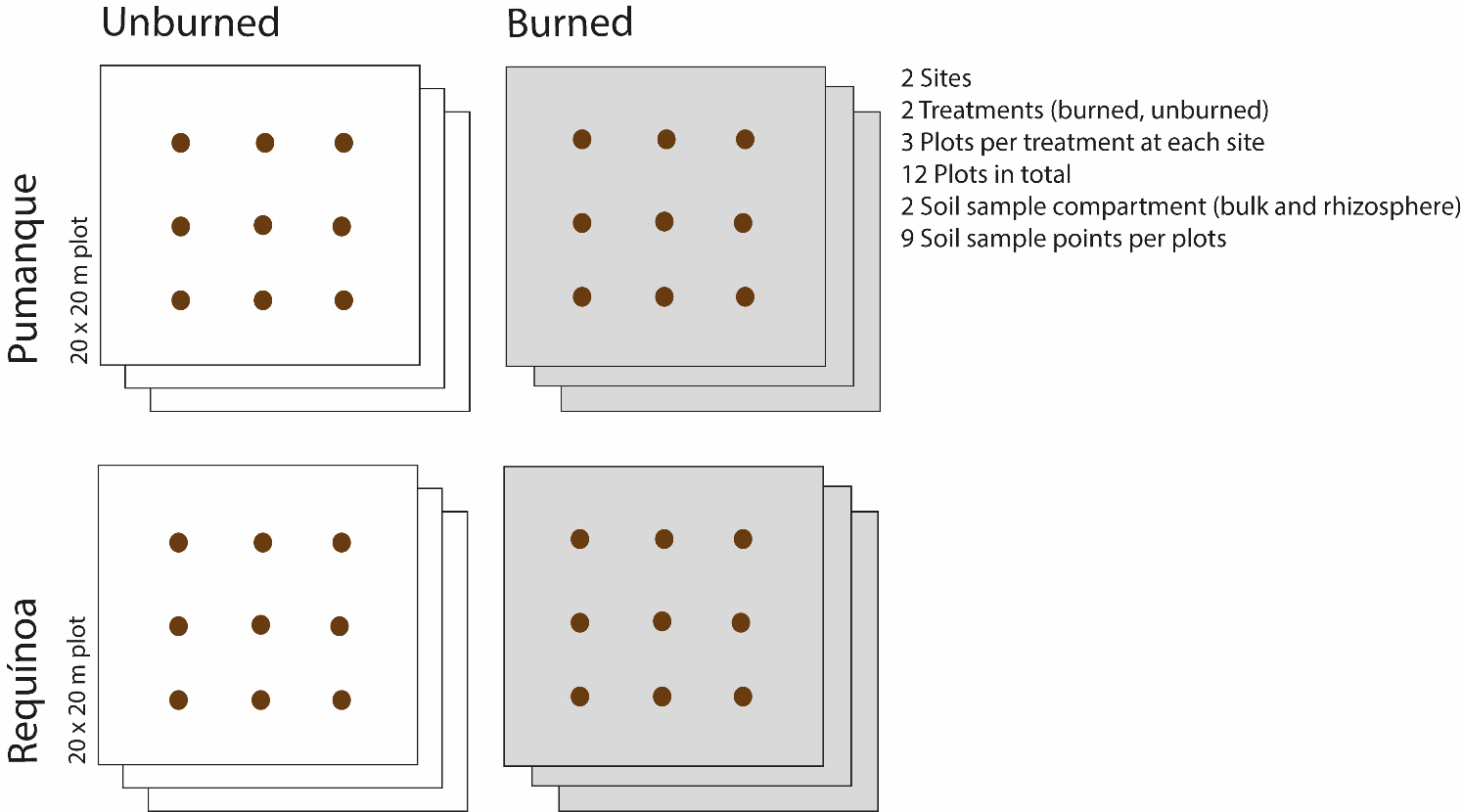


**Supplementary Figure 3**. Experimental design.


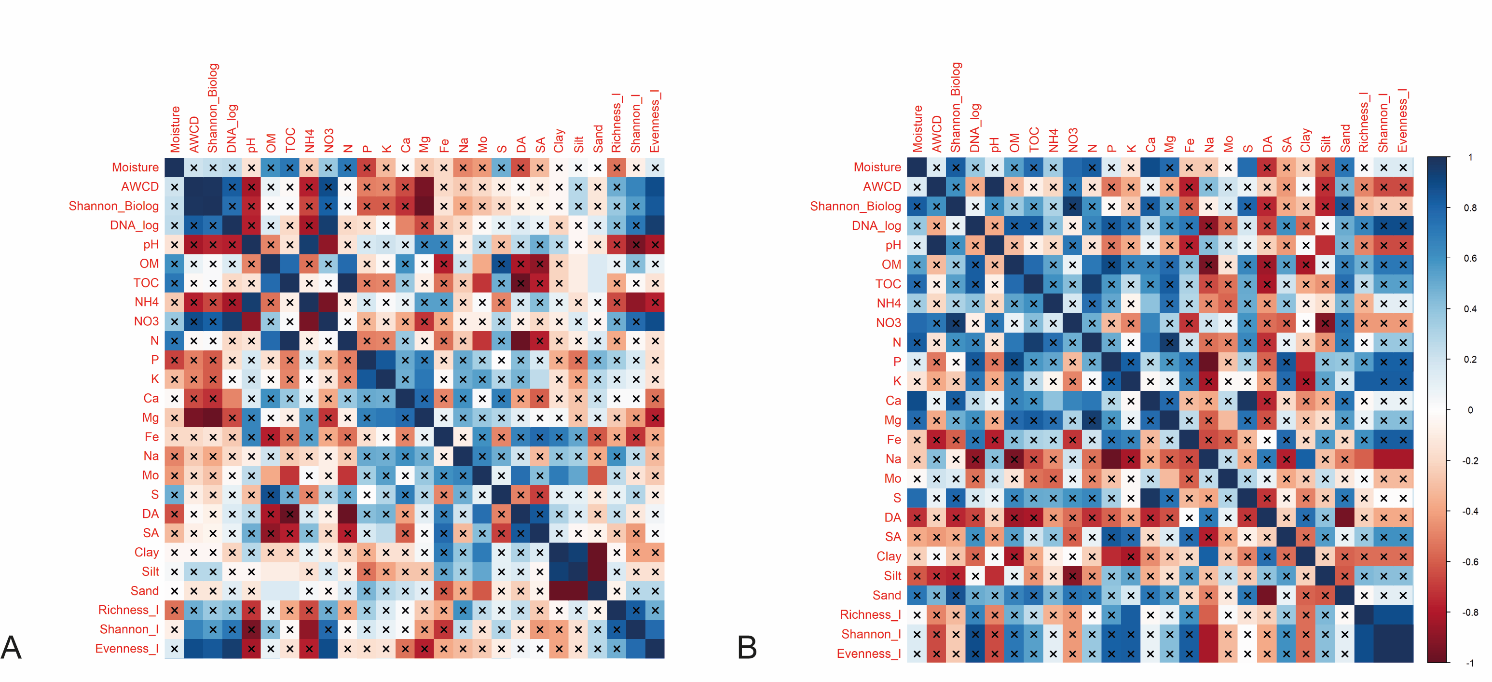


**Supplementary Figure 4.** Spearman correlation matrix for all measured variables from Pumanque (A) and Requínoa (B). Crossed boxed represent non-significant correlations. Red boxes show negative correlation, while blue boxes show positive correlations. Moisture = water content; AWCD = average well development color; Shannon_Biolog = Shannon diversity index obtained from Biolog Ecoplates^TM^; DNA_log = 16S rRNA copy number (log-transformed); OM = soil organic matter; TOC = total organic carbon; NH4 = available ammonium; NO3 = available nitrate; N = total nitrogen; P = total phosphorus; K = total potassium; Ca = total calcium; Mg = total magnesium; Fe = total iron; Na = total sodium; Mo = total molybdenum; S = total sulfur; DA = bulk density; SA = aggregate stability; Clay = clay content; Silt = silt content; Sand = sand content; Richness_I = observed richness of ASVs, Shannon_I = Shannon diversity index obtained from relative abundances of ASVs; Evenness_I = Pielou’s evenness obtained from relative abundances of ASVs.


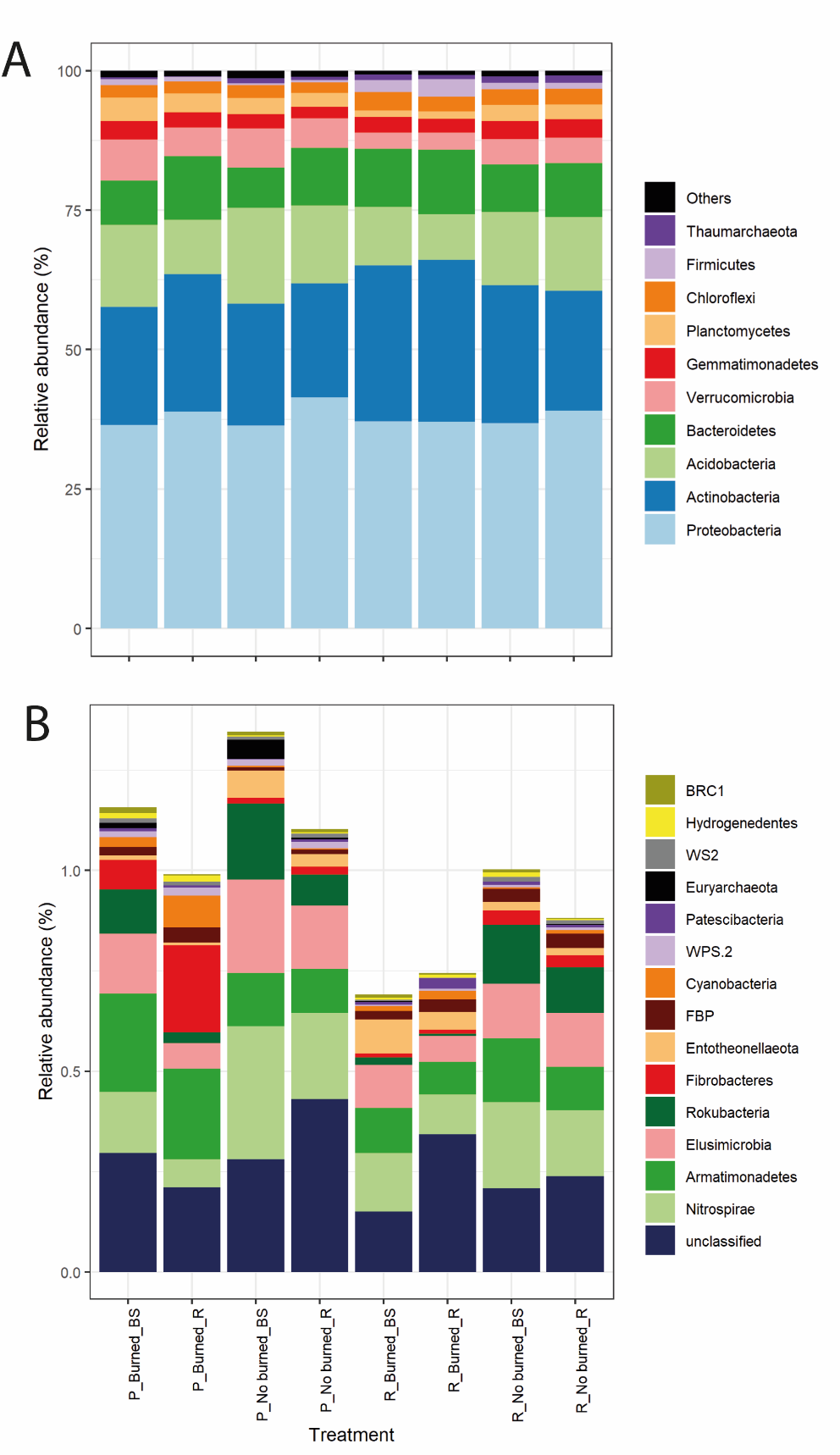


**Supplementary Figure 5.** Mean relative abundances of most frequent (>1% in at least one treatment) (A) and less abundant (< 1%) (B) prokaryotic phyla in burned and unburned bulk and rhizosphere soils from Pumanque and Requínoa. P=Pumanque; R=Requínoa; BS= bulk soil; R=rhizosphere.


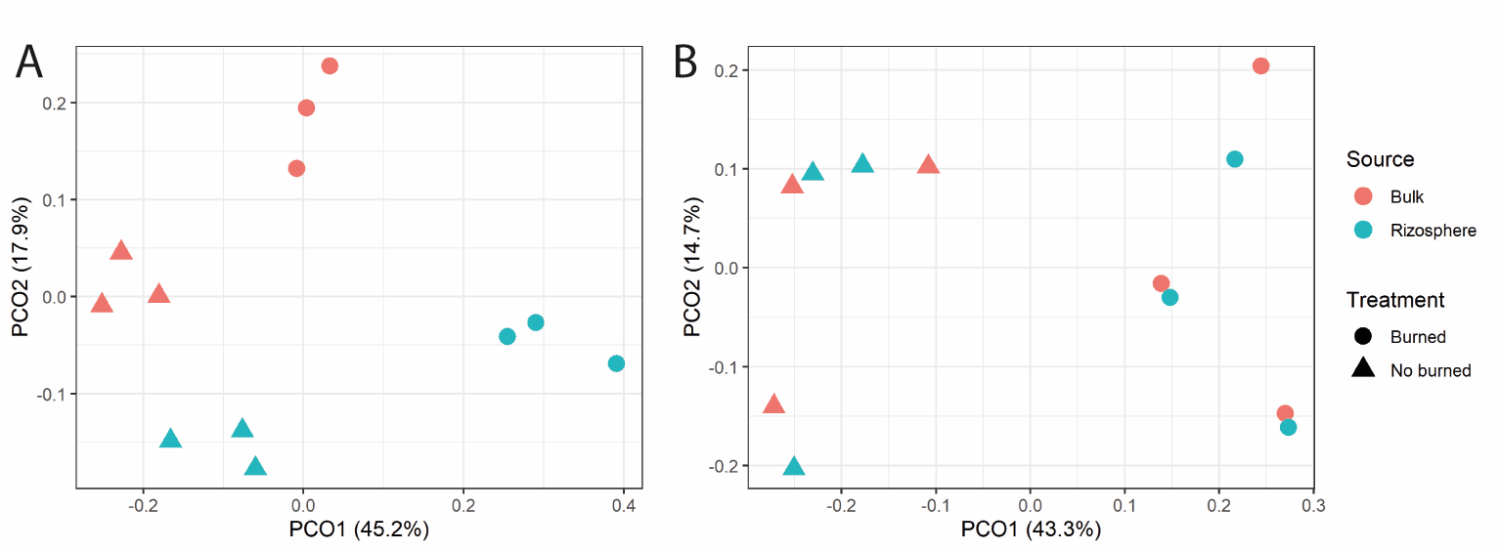


**Supplementary Figure 6.** Beta diversity for prokaryotic communities from burned and unburned bulk and rhizosphere soil samples. Principal coordinate analysis (PCO) based on Bray-Curtis dissimilarities constrained by burned and unburned as well as bulk and rhizosphere soils from Pumanque (A) and Requínoa (B). PERMANOVA was performed by each site independently (Table 1).


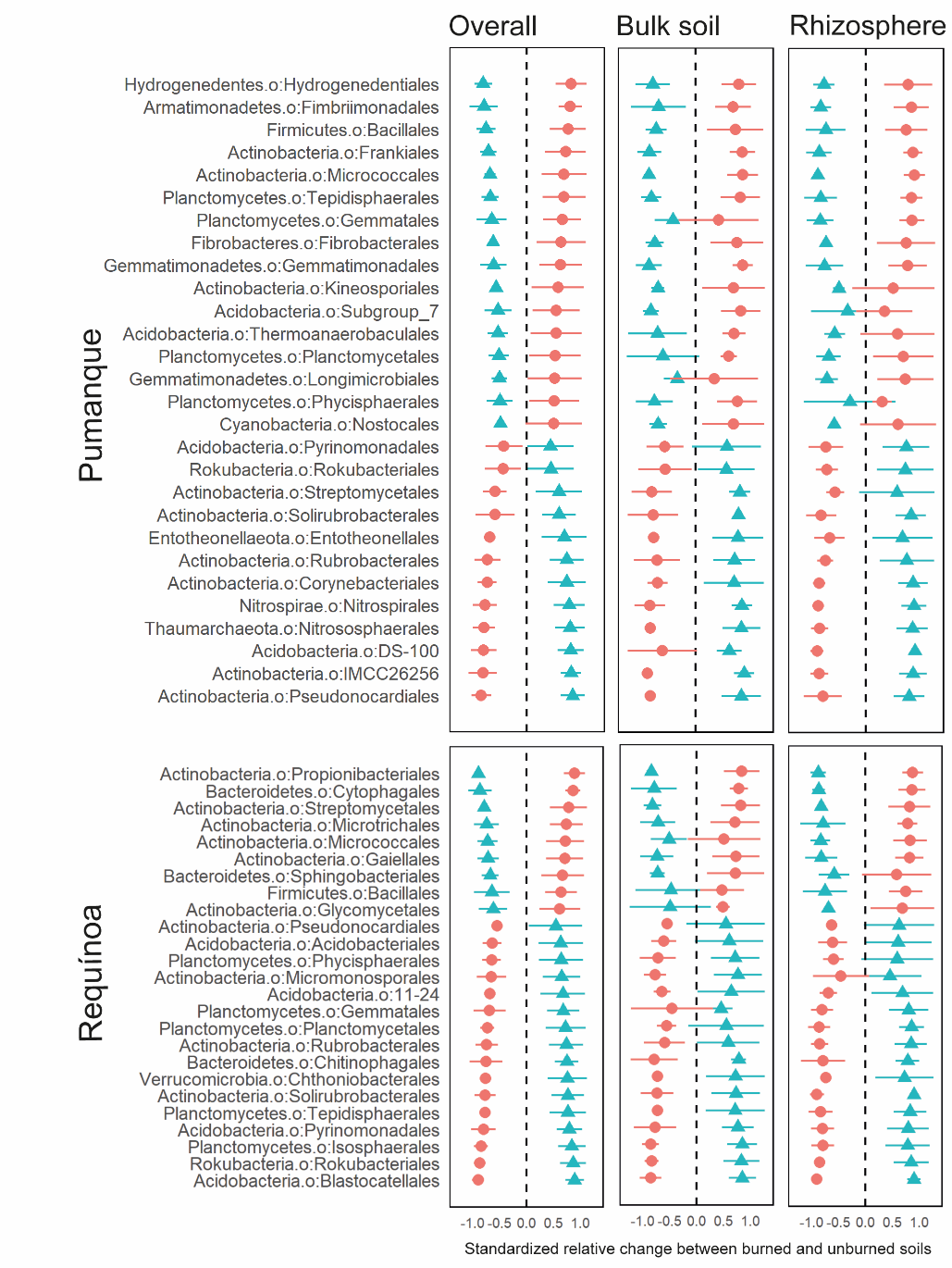


**Supplementary Figure 7**. Relative change (z-transformed) of prokaryote relative abundance, at the Order level, between unburned (blue triangle) and burned (red spot) soils for Pumanque and Requínoa. Only taxa belonging to phyla significantly affected by fires were analyzed, and within these, only orders significantly influenced by fires according to PERMANOVA are shown. The panel to the left shows the relative change in abundance from the overall mean ± s. e. (considering bulk soil and rhizosphere). The middle and left panels show the relative change in abundance for bulk and rhizosphere soils, respectively.


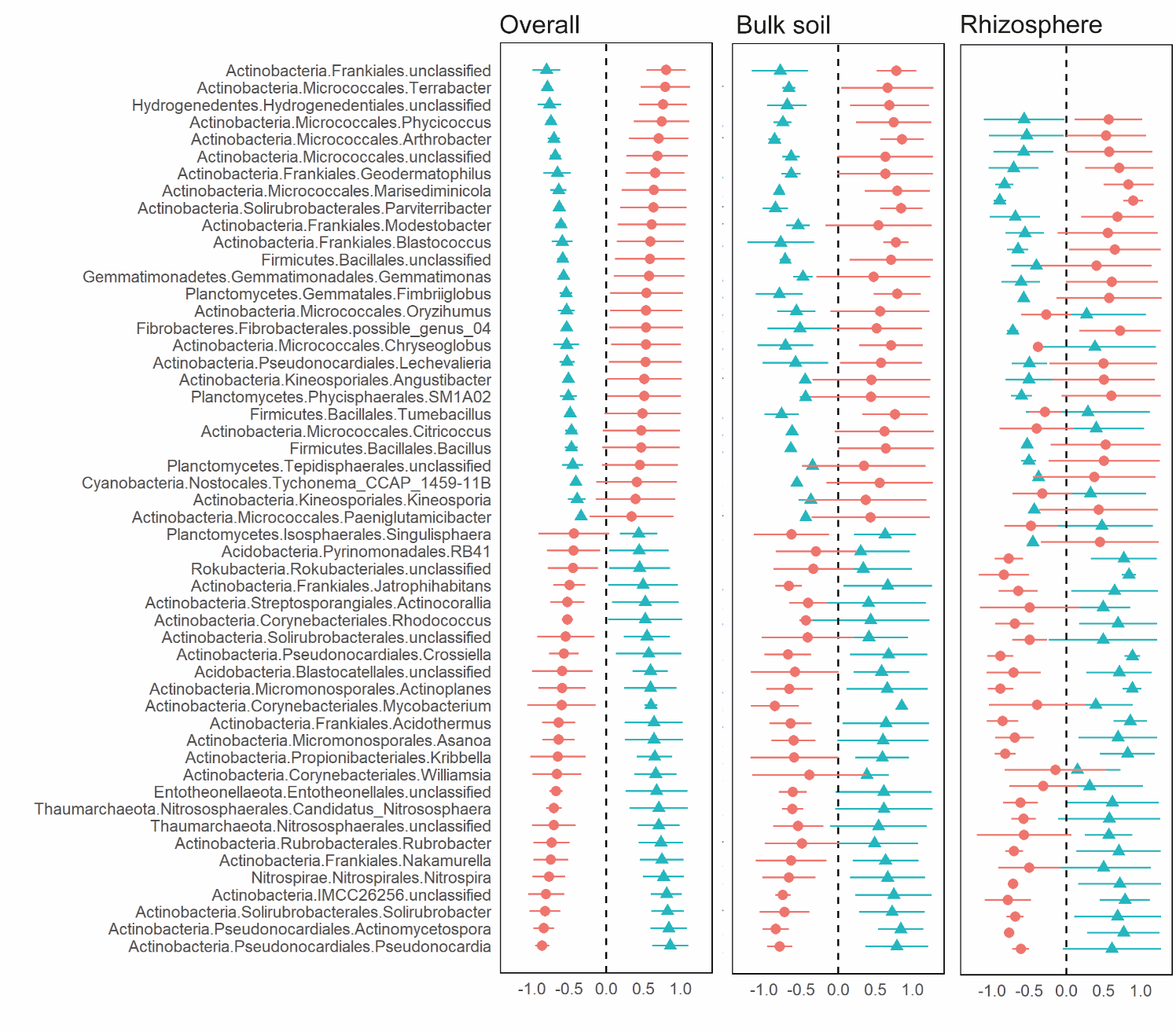


**Supplementary Figure 8**. Relative change (z-transformed) of prokaryote relative abundance, at the Genus level, between unburned (blue triangle) and burned (red spot) soils for Pumanque. Only taxa belonging to Phyla significantly affected by fires were analyzed, and within these, only orders significantly influenced by fires according to PERMANOVA are shown. The panel to the left shows the relative change in abundance from the overall mean ± s.e. (considering bulk soil and rhizosphere). The middle and right panels show the relative change in abundance for Bulk and Rhizosphere soils, respectively.


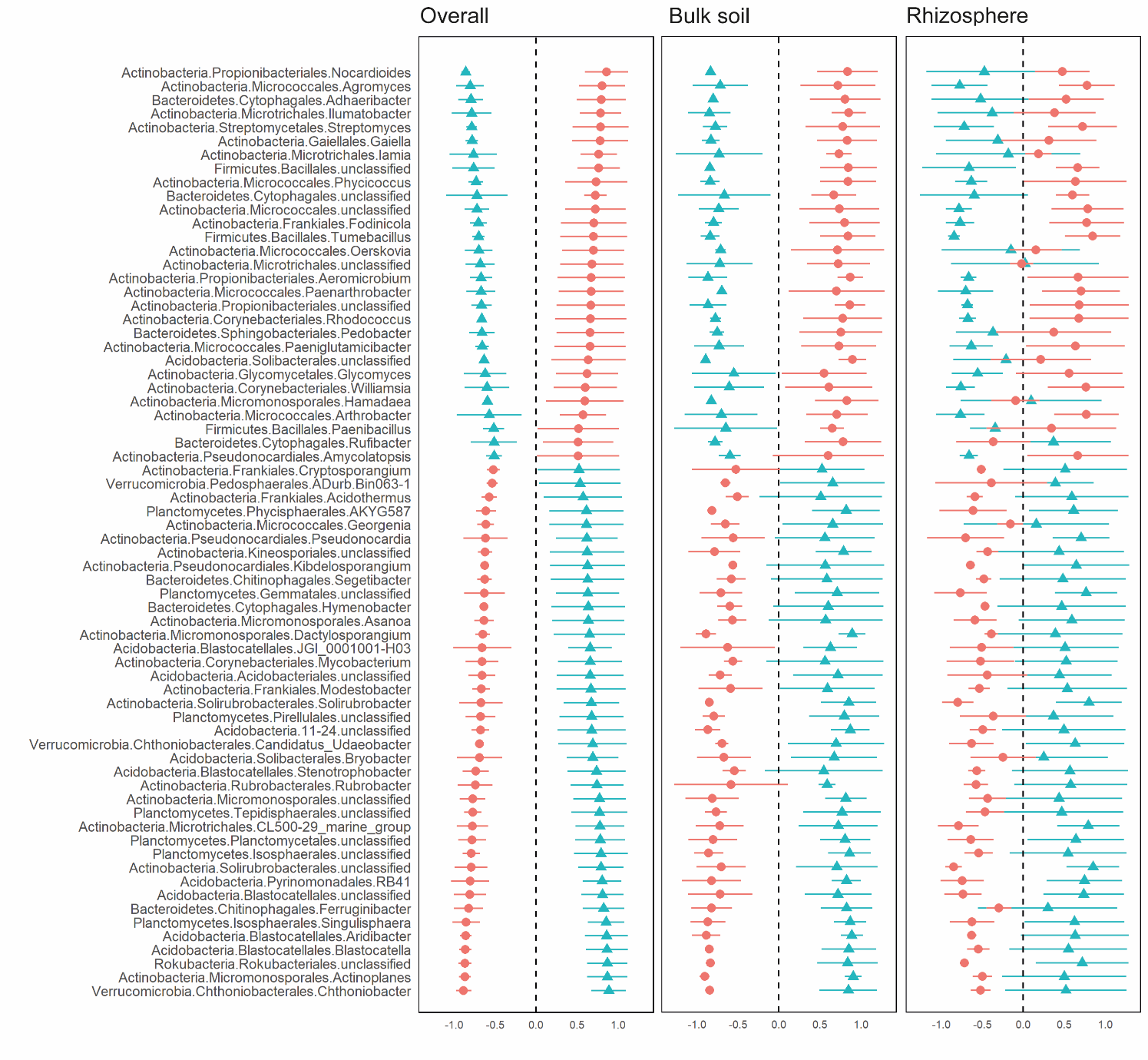


**Supplementary Figure 9** Relative change (z-transformed) of prokaryote relative abundance, at the Genus level, between unburned (blue triangle) and burned (red spot) soils for Requínoa. Only taxa belonging to Phyla significantly affected by fires were analyzed, and within these, only orders significantly influenced by fires according to PERMANOVA are shown. The panel to the left shows the relative change in abundance from the overall mean ± s.e. (considering bulk soil and rhizosphere). The middle and right panels show the relative change in abundance for Bulk and Rhizosphere soils, respectively.

## Supplementary tables

| **Supplementary Table 1**. Soil physicochemical properties of bulk soil samples (mean ± s. e. *n* = 3). | | | | | | | | | |
| --- | --- | --- | --- | --- | --- | --- | --- | --- | --- |
| **Site** | **Treatment** | **pH** | **EC** | **OM** | **TOC** | **N** | **NH_4_** | **NO_3_** | **P** |
| Pumanque | Burned | 7.15 ± 0.13a | 0.18 ± 0.04a | 6.19 ± 1.52a | 4.61 ± 0.75a | 0.29 ± 0.05a | **13.33 ± 2.25a** | **7.33 ± 6.95b** | 0.08 ± 0.01a |
|  | Unburned | 6.86 ± 0.04a | 0.25 ± 0.05a | 9.23 ± 3.81a | 5.44 ± 2.40a | 0.30 ± 0.08a | **6.67 ± 2.73b** | **25.33 ± 2.25a** | 0.08 ± 0.00a |
| Requínoa | Burned | 7.12 ± 0.07A | 0.88 ± 0.15A | 7.11 ± 3.22A | 4.97 ± 2.82A | 0.40 ± 0.22A | 17.00 ± 8.61A | 65.33 ± 53.46A | **0.04 ± 0.01B** |
|  | Unburned | 6.86 ± 0.28A | 0.50 ± 0.27A | 10.72 ± 3.87A | 6.80 ± 2.75A | 0.47 ± 0.13A | 18.67 ± 1.03A | 35.00 ± 20.96A | **0.07 ± 0.01A** |
|  | | | | | | | | | |
| **Site** | **Treatment** | **K** | **Ca** | **Mg** | **Mo** | **S** | **Fe** | **Na** | **BD** |
| Pumanque | Burned | 0.46 ± 0.06a | 1.42 ± 0.66a | 0.38 ± 0.04a | 0.57 ± 0.03a | 0.04 ± 0.01a | 34516 ± 2636a | 175.36 ± 16.83a | 1.14 ± 0.10a |
|  | Unburned | 0.45 ± 0.02a | 1.13 ± 0.20a | 0.35 ± 0.01a | 0.51 ± 0.23a | 0.05 ± 0.01a | 31749 ± 1767a | 174.76 ± 10.91a | 1.11 ± 0.12a |
| Requínoa | Burned | 0.30 ± 0.11A | 1.60 ± 0.55A | 0.65 ± 0.09A | 3.34 ± 1.70A | 0.05 ± 0.02A | **50049 ± 1680B** | **285.08 ± 8.65A** | 1.08 ± 0.13A |
|  | Unburned | 0.51 ± 0.08A | 1.44 ± 0.20A | 0.70 ± 0.02A | 2.08 ± 0.53A | 0.05 ± 0.01A | **58380 ± 3345A** | **199.94 ± 31.93B** | 1.03 ± 0.13A |
|  | | | | | | | | | |
| **Site** | **Treatment** | **AS** | **GMC** | **Clay** | **Silt** | **Sand** |  |  |  |
| Pumanque | Burned | 45.57 ± 10.84a | 4.52 ± 1.89a | 9.33 ± 0.67a | 28.67 ± 2.31a | 62.00 ± 3.46a |  |  |  |
|  | Unburned | 41.89 ± 1.89a | 6.60 ± 1.78a | 6.00 ± 2.31a | 22.67 ± 11.02a | 71.33 ± 14.74a |  |  |  |
| Requínoa | Burned | **30.37 ± 2.80B** | 13.05 ± 2.75A | 24.00 ± 3.06A | 34.67 ± 2.31A | 41.33 ± 6.43A |  |  |  |
|  | Unburned | **37.54 ± 2.04A** | 8.05 ± 3.28A | 16.67 ± 2.91A | 38.67 ± 6.11A | 44.67 ± 7.02A |  |  |  |
| Different lower- and upper-case letters show significant differences for Pumanque and Requínoa, respectively, according to Tukey HSD (p < 0.05). pH (1:2.5 w/v water extract); EC = electrical conductivity) (mS/cm) (1:5 (w/v) water extract; OM = organic matter (%); TOC = total organic carbon (%); N = total N (%); NH_4_ = available ammonium (mg kg^-1^); NO_3_ = available nitrate (mg kg^-1^); P = total phosphorus (%); K = total potassium (%); Ca = total calcium (%); Mg = total magnesium (%); Mo = total molybdenum (%); S = total sulphur (%); Fe = total iron (mg kg^-1^); Na = total sodium (mg kg^-1^); BD = bulk density (g/cc); AS = aggregate stability (%); GWC = gravimetric water content (%); Clay = clay content (%); Silt = silt content (%); Sand = sand content (%). Cases with significant differences are shown in **bold**. | | | | | | | | | |
